# Endodontic Treatment of Type II Dens Invaginatus in a Maxillary Lateral Incisor: A Case Report

**DOI:** 10.1155/2012/153503

**Published:** 2012-11-19

**Authors:** Dilek Helvacioglu-Yigit, Seda Aydemir

**Affiliations:** Department of Endodontics, Faculty of Dentistry, University of Kocaeli, 41190 Kocaeli, Turkey

## Abstract

Dens invaginatus is a developmental anomaly that results in an enamel-lined cavity intruding into the crown or root before the mineralization phase. It typically affects permanent maxillary lateral incisors, central incisors, and premolars. This paper describes the root canal treatment of Oehlers' type II dens invaginatus in maxillary left lateral incisors. A 16-year-old boy presented to the Faculty of Dentistry, University of Kocaeli, to receive his dental treatments. During the caries removal, the pulp was exposed then anendodontic treatment was initiated. Two canals, one of which represented the invagination, were instrumented, irrigated, and then obturated with a lateral condensation technique.

## 1. Introduction 

Dens invaginatus is a developmental anomaly that results in an enamel-lined cavity intruding into the crown or root before the mineralization phase [1, 2]. The literature suggests several aetiologic factors. These are stimulation and subsequent proliferation and ingrowth of cells of the enamel organ into the dental papilla; retardation of a focal group of cells, with those surrounding continuing to proliferate normally during the dental development; external factors like trauma and infection; and also genetic factors [3, 4]. Three invagination categories were proposed by Oehlers [5] to separate the different types of dens invaginatus by the radiographic appearance of invagination: type I: minimal invagination, enamel lined, confined within the crown of the tooth, and does not extend beyond the level of the external amelocemental junction; type II: enamel lined and extends into the pulp chamber but remains within the root canal with no communication with the periodontal ligament; type III: the invagination penetrates through the root, perforating the apical area and having a second foramen in the apical or periodontal area, but there is no immediate communication with the pulp [2, 4]. 

The most frequently affected tooth is the maxillary lateral incisor [2, 3, 6]. In a decreasing order of frequency, other teeth that develop this anomaly are the maxillary central incisors, premolars, canines, and molars [2, 6]. The occurrence of this anomaly in mandibular teeth has been reported in a few cases [4, 7]. The clinical appearance of dens invaginatus varies considerably. The crown of affected teeth can have normal morphology or it can also show unusual forms such as a greater buccolingual dimension, peg-shaped form, barrel-shaped form, conical shapes and talon cusps [1, 2, 8]. A deep foramen caecum might be the first clinical sign indicating the presence of an invaginated tooth. As this area is difficult to access and clean, caries can develop with a subsequent pulp necrosis and apical pathosis [2, 6, 9].

## 2. Case Presentation

**Figure 1:**
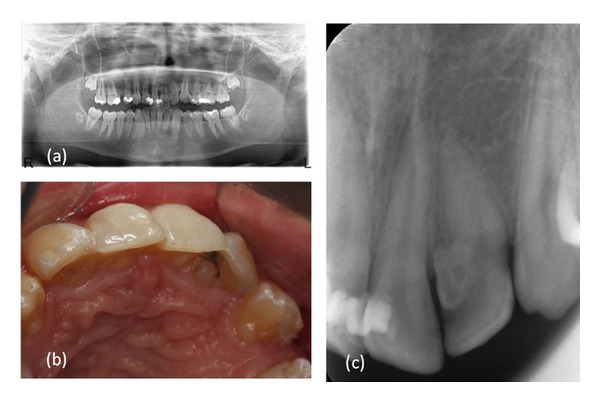
(a) Preoperative panoramic radiograph, (b) preoperative photograph: palatinal view, and (c) preoperative radiograph revealing the maxillary left lateral incisor with an unusual anatomy.

**Figure 2:**
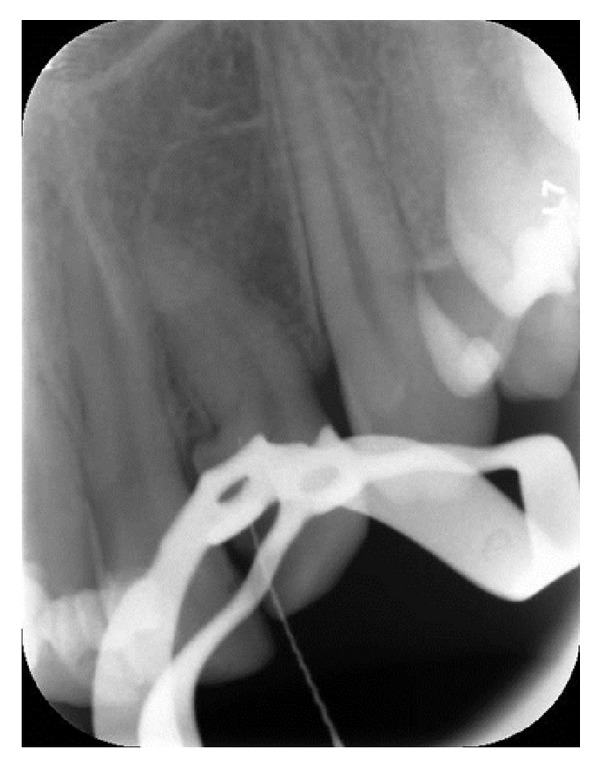
Radiograph showing the fusion of the main canal and invagination.

**Figure 3:**
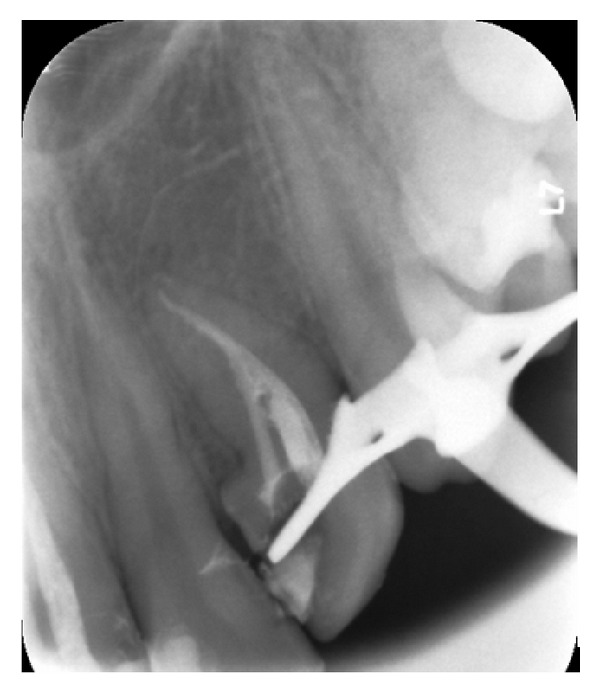
Obturation of root canals.

**Figure 4:**
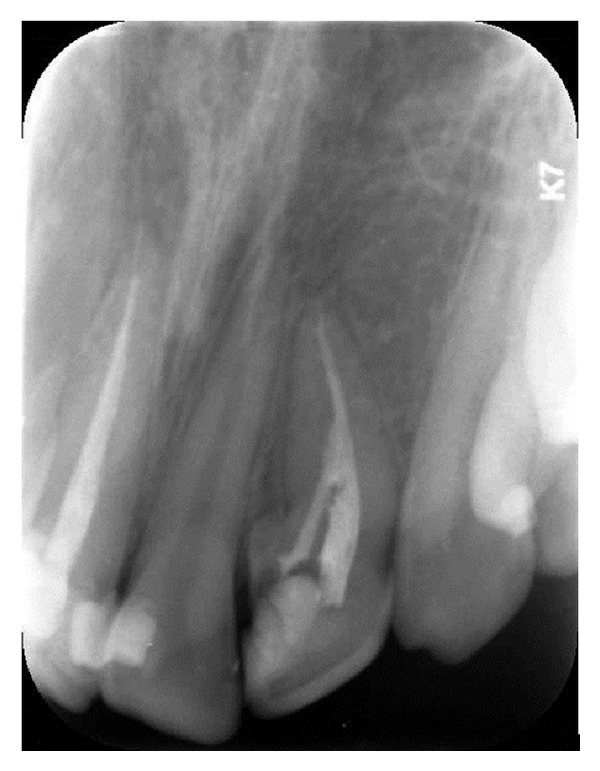
Recall radiograph after 6 months.

A 16-year-old male patient who did not have any problems in his medical history was referred to the Faculty of Dentistry, University of Kocaeli, for his dental treatments. After clinical and radiologic evaluations, we detected caries on the maxillary left lateral incisor which had unusual anatomy (Figures 1(a), 1(b), and 1(c)). The initial periapical radiographic examination revealed that the maxillary left lateral incisor showed an abnormal morphology with an invagination (Oehlers' type II). During the removal of deep dentin caries, the pulp tissue was exposed. The patient was anesthetised, and rubber dam was placed and stabilised using widgets. The main canal and invaginated canal communicated at the middle of the root. The working length was established by a Raypex 5 apex locator (VDWEndodontic Synergy, Munich,Germany). A radiograph showed the fusion of the main canal and invagination (Figure 2). The root canals were prepared with stainless steel H files (Mani Inc., Tochigi, Japan) using a step-back technique. The irrigation was copious throughout with a 2.5% sodium hypochlorite solution, and EDTA (MD-ChelCream, META BIOMED, Chungbuk, the Republic of Korea) was used for chelation. The root canals were dried with paper points and (Precise Dental, Zapopan, Mexico) obturated with a lateral condensation technique with a 0.02 tapered gutta-percha (Diadent, Choongchong Buk Do, the Republic of Korea) and an AH plus (Dentsply De Trey GmbH, Konstanz, Germany) root canal sealer (Figure 3). A two-step self-etch adhesive system (Clearfil SE Bond, Kuraray Medical Inc., Japan) was used in order to perform a restorative treatment. The teeth were restored with a nanofilled resin composite (CLEARFIL MAJESTY Esthetic, Kuraray Medical Inc., Japan). Six months later, the tooth was asymptomatic and all clinical findings were within normal limits (Figure 4).

## 3. Discussion

Several endodontic treatment options of dens invaginatus have been reported, including nonsurgical, surgical, and combined approaches [4, 8, 10–13]. In this paper, the nonsurgical root canal treatment was sufficient since there was no periapical lesion. There are reports of cases in the literature where the pulp tissues were not involved and only the invaginations were cleaned and treated [14, 15]. However, in most cases, the invagination and the pulp tissues can be connected [3, 15]. Cleaning and shaping procedures of the root canal are difficult because the shape of the canal is deformed by the invagination. Like our case in an Oehlers type II dens invagination, the anomaly does not extend all the way to the apex; therefore, access to the apical third of the root canal is less difficult. 

The first difficulty of the cases of dens invaginatus is preparing the access cavity. The root canal debridement of the invagination is difficult, because of the unpredictable shape and narrow access. Stainless steel files using with a chelator such as EDTA and copious irrigation of sodium hypoclorite make the preparation easier allowing thorough debridement of the root canal.

## 4. Conclusion

Dens invaginatus is a rare malformation of the teeth, showing a broad spectrum of morphologic variations in size and form of the crowns and roots. The complex anatomy of these anomalies makes treatment procedures harder. Further followup of these cases should not be neglected to evaluate the treatment success.
